# Analysis of Consanguinity as Risk Factor of Nonsyndromic Cleft Lips with or without Palate

**DOI:** 10.1055/s-0043-1774334

**Published:** 2023-11-23

**Authors:** Yayun Siti Rochmah, Stefani Harumsari, Sandy Christiono, Islamy Rahma Hutami, Siti Fatimah-Muis, Sultana M.H. Faradz

**Affiliations:** 1 Department of Oral and Maxillofacial Surgery, Faculty of Dentistry, Universitas Islam Sultan Agung, Semarang, Indonesia; 2 Department of Medical Biology, Faculty of Medicine, Universitas Islam Sultan Agung, Semarang, Indonesia; 3 Department of Pediatric Dentistry, Faculty of Dentistry, Universitas Islam Sultan Agung, Semarang, Indonesia; 4 Department of Orthodontics, Faculty of Dentistry, Universitas Islam Sultan Agung, Semarang, Indonesia; 5 Department of Nutrition, Faculty of Medicine, Universitas Diponegoro, Semarang, Indonesia; 6 Center for Biomedical Research (CEBIOR), Faculty of Medicine, Universitas Diponegoro, Semarang, Indonesia

**Keywords:** consanguinity, cleft lips, cleft palate

## Abstract

**Objectives** The etiologies of nonsyndromic cleft lips with or without palate (NS CL/P) are multifactorial, which include consanguineous marriages. The incidence of NS CL/P is relatively high in Indonesia notably in one of Indonesia's tribes whose members frequently marry close cousins. Thus, the purpose of this study is to analysis consanguinity as risk factor of NS CL/P in Sasak tribe, East Lombok, Indonesia

**Materials and Methods** An observational analysis was made of a collected database of NS CL/P patients treated in social services in regency hospital of Dr. Soejono Selong, East Lombok, Indonesia. Demographic data such as age, gender, address (urban/rural), parent's education, presence or absence of consanguinity, type of clefts, and a three-generation pedigree were collected by interview and hospital medical record. Before analysis, patient information was anonymized and deidentified. From 2016 to 2018, each of 100 cleft and normal subjects with their Sasak parent were audited. The risk factors were analyzed statistically using odds ratio (OR) and chi-squared test.

**Results** Consanguineous marriages identified 54 cases (54%), and 10 cases (10%) out of a total each 100 NS CL/P and controls, respectively. The majority of consanguinity (53.7%) was discovered in marriages between first cousins. NS CL/P cases were statistically linked ( *p* = 0.00) with consanguineous marriages (OR: 10; 95% confidence interval: 1.6–3.1); in which the most prevalent case is unilateral cleft lips.

**Conclusion** Consanguineous marriage increases the risk of NS CL/P in Sasak tribe, East Lombok, Indonesia. The development of strategies to educate communities on the impacts of culture-consanguineous marriage is required. The genetic inheritance from their ancestor may be responsible for the increased incidence of NS CL/P.

## Introduction

Nonsyndromic cleft lips or palate (NS CL/P) is a maxillofacial congenital deformity. In Indonesia, the incidence of NS CL/P is relatively high. From 2011 to 2015, the Lip and Palate Cleft Foundation in West Java handled 1,596 NS CL/P patients. The greatest rate is 50.53% for cleft lip and palate (CL/P), followed by 24.42% for cleft lip (CL). Approximately 55.95% of the population consisted of males with the majority of region of cleft on the left side. 1

NS CL/P is caused by multiple factors, 2 one of which is consanguineous or relative marriage. 3 In numerous tribes throughout the world, consanguinity is prevalent, similar to the traditions of various Indonesian tribes, including the Sasak tribe of East Lombok, Indonesia. The Sasak tribe has a practice of elopement ( *merariq* ) in which the guy abducts the woman and then proposes to her. This elopement custom is typically practiced within the context of communal groupings that still include a single family member. The Sasak tribe constitutes around 90% of Lombok's overall population. There is a word called “limitation of mate” that refers to parents who limit their child's choice of spouse on the basis that marriage with their own relatives is preferable to marriage with outside relatives. The custom of marrying within the Sasak tribes is so prevalent with the belief that family ties will not weaken. 4

The Sasak tribe desires to implement the practice of marrying relatives or weddings among family members in order to preserve the integrity of a tribal group's kinship links (kin group endogamy). Consanguinity allows autosomal recessive genes to be expressed due to mutations, resulting in phenotypic defects such as cleft lip or palate, according to genetic theory. In a 2015 to 2018 study conducted in an Arab country, palatal cleft data were shown to be substantially different with consanguinity ( *p* = 0.047, odds ratio (OR): 2.5, 95% confidence interval: 1–6.46, to be exact). 5 Approximately 62.8% of the research conducted in Pakistan revealed a correlation between kin marriage and NS CL/P. Marriage to a relative is a significant risk factor for congenital anomalies, including NS CL/P with an OR of 2. 6 Other research have also identified kinship marriage as a risk factor with an OR of 2.5. 7 This indicates that having children with a complicated cleft lip is more prevalent, as it occurs in cases of mating between relatives. To date, the number of NS CL/P cases in Lombok has a prevalence of cleft lip above the national prevalence. 8 This study was designed to determine whether consanguinity is a risk factor for NS CL/P among the Sasak tribe of East Lombok.

## Materials and Methods

### Patients Criteria

This study's population and samples were taken from the Sasak tribe in East Lombok, Indonesia, which participated in the local community health service. Cleft subjects consisted of all patients undergoing oral maxillofacial and plastic surgery with medical records. Controls were obtained from children participating in the local community health service, who have been matched with age, regardless of gender, and have a complete parental background. Each of 100 cleft and normal subjects with their Sasak parent was audited between 2016 and 2018. These patient medical records and information were made anonymous and reidentified. In Dr. Soejono Selong's hospital situated in East Lombok, an observational study was undertaken by collecting medical records form of all cleft patients undergoing oral maxillofacial and plastic surgery and conducting questionnaire interviews including pedigree with their mothers. All methods of the experiment were approved by the Ethical Committee at Faculty of Medicine, Universitas Diponegoro, No.023/EC/FK-RSDK/2016.

### Experimental Procedure

Subjects with cleft and controls completed the required questionnaire after talking with their mothers. The questionnaire data include basic demographic, marriage histories, pedigree construction would then be added to the database and processed. The data was extracted from the medical records of oral and maxillofacial surgery and plastic surgery patients. The hospital records contained basic demographic information including age, gender, address (urban/rural), and parental education. By Labial-Alveolar-Hard Palate-Soft Palate-Aleveolar-Labial categorization, the cleft type was recorded using a basic descriptive description. 9 CL was further categorized as unilateral and bilateral, CP as partial cleft palate, and CLP as complete cleft lips and palate. The objective of a questionnaire interview with the parent was to construct the three-generation pedigree by collecting information on parental consanguinity.

### Statistical Analysis

The data analysis was performed with the SPSS program (SPSS Inc., Chicago, Illinois, United States). The risk factors were analyzed statistically using OR and Chi-square test.

## Results

### The Distribution of Deformities in Sasak Tribe

There were 100 patients with NS CL/P, 42 men and 58 females. Fifty-four out of the 100 cleft patients had parental consanguinity history. While 100 normal subjects categorized as the control group, 10 parents were found with a history of consanguinity. This result showed that many Sasak tribe with cleft had a history of consanguinity. Meanwhile, based on clinical conditions, the highest incidence of unilateral cleft lips was among 22 female patients ( Table 1 ).

**Table 1:** Distribution of cleft deformities

Gender (n)	CL (%)		CP (%)		CLP (%)
	Unilateral	Bilateral	Partial	Complete	
Male: 42 (42%)	21 (48.8%)	10 (52.6%)	3 (30%)	6 (30%)	2 (25%)
Female: 58 (58%)	22 (51.2%)	9 (47.4%)	7 (70%)	14 (70%)	6 (75%)
	43 (43%)	19 (19%)	10 (10%)	20 (20%)	8 (8%)
Total: 100	62 (62%)		30 (30%)		8 (8%)

Abbreviations: CL, cleft lips; CLP, cleft lips and palate; CP, cleft palate.

### Risk Analysis of Consanguinity in Sasak Tribe

From the total NS CL/P, there was 54 patients with history of consanguinity. The risk analysis for the Sasak tribe increased three times higher when compared to those without cleft patient in OR chi-squared analysis ( Table 2 ).

**Table 2:** Analysis of consanguinity in cases and controls

**Consanguinity**	**NS CL/P**		***p*** **-Value**	**OR (95% CI)**
	**Yes (case)**	**No (control)**	0.000	10 (1.6–3.1)
Yes	54 (54%)	10 (10%)		
No	46 (46%)	90 (90%)		
Total	100 (100%)	100 (100%)		

Abbreviations: CI, confidence interval; NS CL/P, nonsyndromic cleft lips with or without palate; OR, odds ratio.

### The Prevalent Type of Consanguinity Causing NS CL/P

**Fig. 1:**
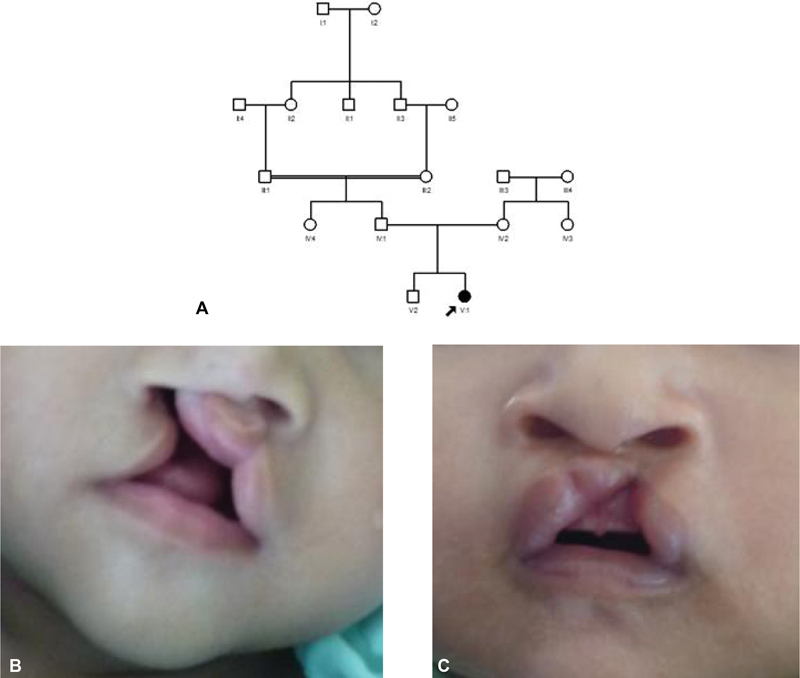
The overview of consanguinity and nonconsanguinity case in minor patients. ( **A** ) Pedigree of the cleft in three-generation pedigree. ( **B** , **C** ). The typical of the with or without palate with (V1) and without (V2) consanguinity case history. The most severe case was observed in a patient with a history of consanguinity.

Consanguinity data obtained from three generation pedigree can be seen in Fig. 1 . According to all the data on NS CL/P NS subjects with a history of consanguinity, first cousins were the most prevalent of consanguinity ( Table 3 ).

**Table 3:** Type of consanguinity of patients with NS CL/P

Mode of consanguinity	CL (%)		CP (%)		CLP (%)	Total
	Unilateral	Bilateral	Partial	Complete		
First cousin	17 (31.5%)	3 (5.5%)	2 (3.7%)	4 (7.4%)	3 (5.5%)	29 (53.7%)
Second cousins	2 (3.7%)	0	1 (1.9%)	0	1 (1.9%)	4 (7.4%)
Double first cousins	4 (7.4%)	1 (1.9%)	0	1 (1.9%)	1 (1.9%)	7 (13%)
First cousin one removed	3 (5.5%)	1(1.9%)	1 (1.9%)	0	1 (1.9%)	6 (11%)
Double second cousin	4 (7.4%)	1 (1.9%)	0	1 (1.9%)	2 (3.7%)	8 (14.9%)
Total	30 (55.6%)	6 (11%)	4 (7.5%)	6 (11%)	8 (14.9%)	54 (100%)

Abbreviations: CL, cleft lips; CLP, cleft lips and palate; CP, cleft palate; NS CL/P, nonsyndromic cleft lips with or without palate.

## Discussion

This study showed that CL predominated 62%, followed by CP 30% and CLP 8%. CP is more prevalent in males but cleft lip and palate and cleft labial are more prevalent in females. In relation to gender, the chance of acquiring cleft was 58% for females. There is currently no commonly recognized reason for these sex differences; nevertheless, they can be explained by changes in the timing of crucial stages of craniofacial development in female and male embryos. 10 Based on gender differences in the timing of these important embryological stages with respect to the orofacial clefts (OFC) categories in general and the OFC sub phenotypes in particular, there has been essentially no emphasis in the literature yet, and little is known about these disparities. It also raises the risk for CP and may explain the female prevalence of CP, as the female palatal shelves become horizontal approximately a week later than in male embryos. 11 This is contrary to results from studies conducted in Brazil, which indicate that the incidence of NS CL/P is 2:1 higher in males than in female. 12 The exposure of the CLP in females with relative CP was less than in males. Genetic and molecular research is important for a better understanding of the distinctions between mouth cleft types and sex. Depending on the location of the cleft, the right side is the most frequent. 13 According to research conducted in India, left-sided unilateral NS CL/P clefts in children were more prevalent than their right-sided counterparts. 14

Given that the right-sided fetal head arteries leave the aortic arch closer to the heart, it has been hypothesized that the right side may have a better hemodynamic perfusion. One of the risk factors for NS CB/P diseases is consanguinity. In this study, data was collected from 54 (54%) of 100 NS CL/P cases with a history of married relatives in the Sasak tribe. The difference between the CL/P and control groups was statistically significant, indicating that people with consanguinity had an increased chance of having children with CL/P. The majority of consanguineous relationships include first cousins (53.7%). This is consistent with the obtained data, specifically in populations of North Africa, West Asia, and South India, where consanguineous marriages are culturally and socially favored and account for 20 to 50% of partnerships, roughly one-third of all marriages involve first cousins 15 and another retrospective research investigated the characteristics of parental consanguinity in multi-ethnic groups that more than 76% of all consanguineous unions were categorized as first cousins. 16 The vast majority of consanguineous marriages in our society continues to be between first and second cousins. Additionally, consanguinity has been linked to facial clefts and other congenital malformations. 17 But in this research, the cleft cases were nonsyndromic.

Almost 82% of neonates born with CL/P were born of consanguineous marriages, and those newborns were 4.5 times more likely to have OFC than those born of nonconsanguineous marriages. 18 It is possible for consanguineous marriages to induce autosomal recessive disorders, including extremely rare or new syndromes, thereby increasing public awareness of the associated risks. Consanguinity also offers certain social advantages observed in rural areas, such as the facilitation of increased female autonomy, marriage arrangements, more stable marital relationships, less domestic violence, greater compatibility with in-laws, economic benefits of reduced dowry, lower divorce rates, and the preservation of any landholding. 14 17 Genetically, consanguinity is a result of the reduction in genetic diversity generated by meiosis, which occurred due to the smaller number of close relatives. Due to the fact that 99.6 to 99.9% of the human genome is shared by all people, consanguineous marriages have had a negligible effect on sequencing. 19 20 If two siblings gave birth, the child will have two grandparents rather than four. Under these circumstances, the child is more likely to inherit two copies of a harmful recessive gene (allele). 21

Homozygosity rates are higher in offspring of consanguineous parents. Loss of function (LoF) mutations that result in the complete inactivation or malfunctioning of genes are likely to arise in autozygous regions of their genome. Through the study of consanguineous offspring with clinical characteristics, it is feasible to identify the mutations responsible for a disease. Nevertheless, the bulk of genes in the human genome either do not correspond to any known disorders or have unknown roles. This is likely the result of undiscovered homozygous LoF mutations in outbred populations, which are the primary focus of massive sequencing research. Many genes in the genome, however only several genes related to the unique or well-defined cleft phenotype. 21 In this study, there were 43 participants with unilateral CL ( Table 1 ), and as many as 30 participants had a history of consanguineous marriages ( Table 3 ). This demonstrated that consanguinity was responsible for the majority of NS CL/P instances in the Sasak tribe. This study predicted consanguinity via pedigree (family-free) analysis, as depicted in Fig. 1 , so that Sasak tribe consanguineous marriages could be investigated. Every built relationship prediction model must account for genotyping errors. 22

**Fig. 2:**
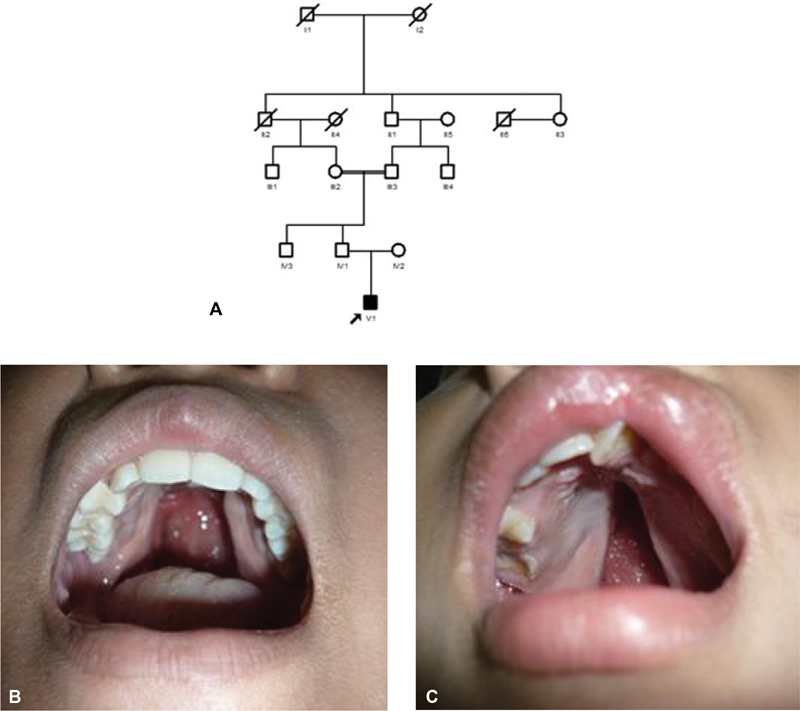
The overview of consanguinity and nonconsanguinity case in adult patients. ( **A** ) Pedigree of the cleft in three-generation pedigree. ( **B** , **C** ). The typical of the with or without palate with (V1) and without consanguinity case history. The most severe case was observed in a patient with a history of consanguinity.

Marriage between first cousins was the most common cause of unilateral cleft lip (UCL) (31.5%), whereas uncle–niece marriage was the least common ( Table 3 ). In this study, there were disparities between consanguinity and nonconsanguinity regarding phenotype cleft. There was the relationship between the type of marriage and the phenotypic state, as shown in Fig. 1A unilateral cleft lips with consanguinity history, Fig. 1B three-generation pedigree tree, and Fig. 1C cleft lips nonconsanguinity history. Clinically, unilateral cleft lips with consanguinity history are more severe than unilateral cleft lips nonconsanguinity history. Clinical differences are also seen in Fig. 2 ; cleft palate with consanguinity history ( Fig. 2A ) is more severe than cleft palate not associated with consanguinity ( Fig. 2C ). The phenotypic was more severe in patients with a history of consanguinity than nonconsanguinity cases. This is supported by the hypothesis that autosomal recessive gene mutations are inherited from a common ancestor. 23

Individuals born of consanguineous marriages have homozygous genomic segments because they received identical ancestral segments from both parents. Consanguinity increases the likelihood of mating between heterozygotes carrying the same recessive mutant gene, but has little effect on the allele frequencies of common diseases. 24 This is because, for prevalent recessive disorders, there is a substantial probability that unrelated spouses may carry the faulty gene and pass it on to their offspring. As a result, it is anticipated that the probability of birth abnormalities in the offspring of first-cousin marriages will be significantly higher than in nonconsanguineous marriages, particularly for uncommon autosomal recessive disease genes. 23 25

Mothers with homozygous genotype polymorphism would pass on offspring with a more severe phenotype than heterozygous mothers. 26 This discrepancy in phenotype may be attributable to the multiple causes of NS CL/P, as well as the effects of maternal folate consumption during pregnancy on embryogenesis and fetal phenotype regulation due to epigenetic mechanisms. 27 28 With The advanced technology in molecular biology such as next-generation sequencing for trio analysis (child, mother, and father) probably can explain the inherited recessive alleles. Therefore, further study to analyze possible pathogenic variants in CLP cases is warranted. Education to the community about the effect of consanguineous married should be proposed to the local government and social organization.
